# Inhibition of CK2α Down-Regulates Hedgehog/Gli Signaling Leading to a Reduction of a Stem-Like Side Population in Human Lung Cancer Cells

**DOI:** 10.1371/journal.pone.0038996

**Published:** 2012-06-29

**Authors:** Shulin Zhang, Yucheng Wang, Jian-Hua Mao, David Hsieh, Il-Jin Kim, Li-Min Hu, Zhidong Xu, Hao Long, David M. Jablons, Liang You

**Affiliations:** 1 Thoracic Oncology Laboratory, Department of Surgery, Helen Diller Family Comprehensive Cancer Center, University of California San Francisco, San Francisco, California, United States of America; 2 Lung Cancer Institute, Sun Yat-sen University, Guangzhou, People's Republic of China; 3 Department of Surgical Oncology, The Third Affiliated Hospital of Guangzhou Medical University, Guangzhou, People's Republic of China; 4 Department of Surgery, Helen Diller Family Comprehensive Cancer Center, University of California San Francisco, San Francisco, California, United States of America; 5 Life Sciences Division, Lawrence Berkeley National Laboratory, Berkeley, California, United States of America; 6 Department of Obstertrics and Gynecology, University of California San Francisco, San Francisco, California, United States of America; Mayo Clinic College of Medicine, United States of America

## Abstract

Protein kinase CK2 is frequently elevated in a variety of human cancers. The Hedgehog (Hh) signaling pathway has been implicated in stem cell maintenance, and its aberrant activation has been indicated in several types of cancer, including lung cancer. In this study, we show that CK2 is positively involved in Hh/Gli signaling in lung cancer cell lines A549 and H1299. First, we found a correlation between CK2α and Gli1 mRNA levels in 100 primary lung cancer tissues. Down-regulation of Gli1 expression and transcriptional activity were demonstrated after the silencing of CK2α in lung cancer cells. In addition, CK2α siRNA down-regulated the expression of Hh target genes. Furthermore, two small-molecule CK2α inhibitors led to a dose-dependent inhibition of Gli1 expression and transcriptional activity in lung cancer cells. Reversely, forced over-expression of CK2α resulted in an increase both in Gli1 expression and transcriptional activity in A549 cells. Finally, the inhibition of Hh/Gli by CK2α siRNA led to a reduction of a cancer stem cell-like side population that shows higher ABCG2 expression level. Thus, we report that the inhibition of CK2α down-regulates Hh/Gli signaling and subsequently reduces stem-like side population in human lung cancer cells.

## Introduction

Protein kinase CK2 (formerly known as casein kinase II) is a highly conserved serine/threonine kinase that phosphorylates more than 300 proteins [1]. CK2 has a heterotetrameric structure consisting of two catalytic subunits (42-kDa α or 38-kDa α’) and the regulatory subunit (28-kDa β), forming the configurations α2β2, αα’β2 and α’2β2. CK2 is a multifunctional protein kinase [2], that has been shown to be involved in nearly every aspect of cell proliferation and survival [3], [4], [5]. The level of CK2α expression is tightly regulated in normal cells [6], and increased CK2α level and activity has been consistently observed in a variety of human cancers [7], [8], [9]. For instance, the high level and/or nuclear localization of CK2α is a marker of poor prognosis for patients with acute myeloid leukemia, chronic lymphocytic leukemia, prostate cancer and gastric cancer [10], [11], [12], [13]. CK2 also affects several cell signaling pathways, including PI3K, NFkB and Wnt [6], [14], [15].

The Hedgehog (Hh) family of secreted proteins, which consists of Sonic, Indian and Desert Hedgehog, plays important roles in mammalian development and in stem cell maintenance [16], [17]. Activation of the Hh pathway is initiated at the cell surface by the Hh ligand binding to its receptor Patched (Ptc), resulting in derepression of the effector protein, a G-protein-coupled receptor, Smoothened (Smo) [18]. Ultimately, Smo activates the Gli family of transcription factors and target genes. There are three Gli proteins in humans: Gli1 serves to activate Hh target genes, Gli2 acts both as activator and repressor of Hh target genes, while Gli3 acts as a repressor of Hh target genes [19], [20]. Deregulation of Hh/Gli signaling is implicated as an initiating or maintaining factor in the progression of various cancers, including basal cell carcinomas, medulloblastomas, leukemia, lung, gastrointestinal, lung, ovarian, breast and prostate cancers [19], [21]. For instance, the Gli1 gene is amplified in human glioma and activated in basal cell carcinoma [22], [23], [24]. Transgenic over-expression of Gli1 in mice leads to the development of basal cell carcinoma [25]. Gli1 activation has been demonstrated in non-small cell lung cancer (NSCLC) cells and tissues [26].

Direct evidence that Hh/Gli signaling plays an important role in cancer stem cells (CSCs) derives from a series of studies in different tumor types [21]. Recent studies have indicated that ATP-binding cassette transporter member 2 of G family protein (ABCG2) is a direct transcriptional target of Hh/Gli signaling [27]. A subpopulation, named side population (SP) with higher ABCG2 expression level in human cancer cells including lung cancer A549 cells, showed series of CSCs’ characteristics [28], [29], [30]. Several lines of recent evidence suggest that hedgehog signaling regulates stem-like side population in human lung cancer cells. For instance, the side population of lung cancer cell line H460 preferentially expresses ABCG2 and SMO, a critical mediator of the hedgehog signaling. Cyclopamine, a natural hedgehog pathway inhibitor, greatly inhibits cell-cycle progression and cell proliferation of H460 cell line [30]. Cyclopamine also reduced side population in human cancer cells [31]. Furthermore, Hedgehog pathway inhibitor GDC-0449, a FDA approved drug for treatment of metastatic basal cell carcinoma, effectively reduced cell growth in human lung cancer cell lines. The effect is mediated by the inhibition of stem-like side population [32].

To date, there is no evidence for the relationship between CK2 and Hh/Gli signaling in mammalian cells. To investigate whether CK2 is involved in the Hh pathway in human lung cancer cells, we tested the activity of Gli1 after CK2 inhibition.

## Results

### CK2α and Gli1 Genes are Activated and Correlated in Human NSCLC

**Figure 1:**
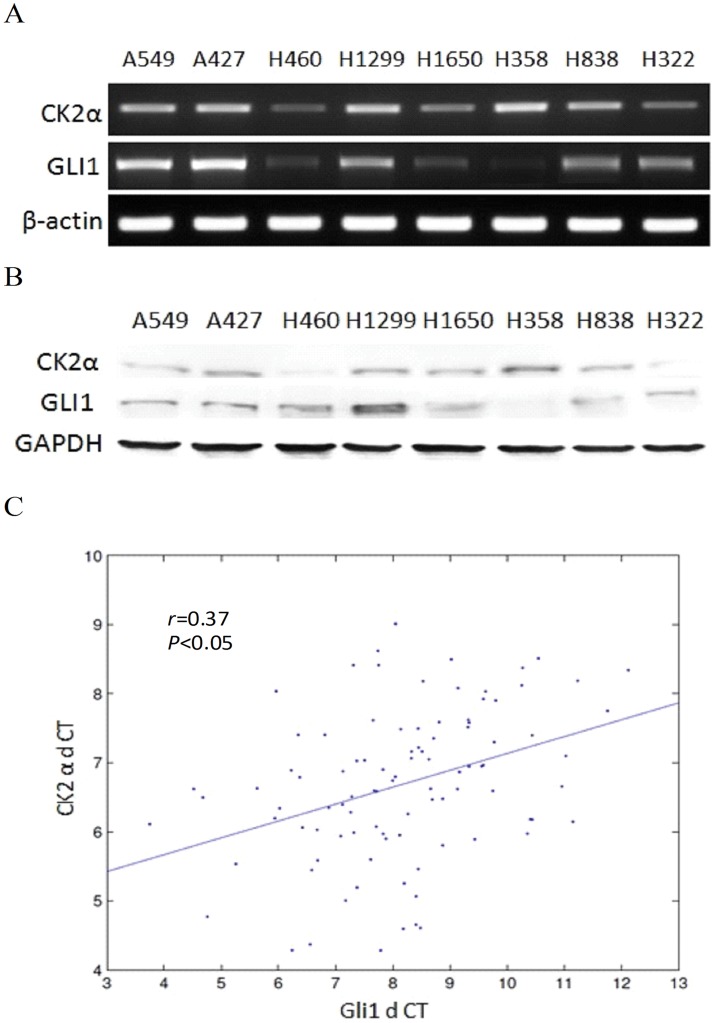
Over-Expression and Correlation of CK2α and Gli1 Genes in NSCLC. (A) RT-PCR. (B) Western blot. The CK2α gene was activated in the eight NSCLC cell lines examined (A549, A427, H460, H1299, H1650, H358, H838 and H322), and the Gli1 gene was expressed in all the cell lines except H358. (C) Linear correlation curve of CK2α and Gli1 mRNA levels. A mild correlation (r = 0.37, *P*<0.05) was shown in linear correlation analysis by SPSS. Primary NCSCL tissues from patients undergoing resection were collected at the time of surgery and immediately snap-frozen in liquid nitrogen. These tissue samples were kept at –170°C in a liquid nitrogen freezer before use.

Both CK2α and Gli1 genes have been shown to be over-expressed in a variety of cancers, including lung cancer [26], [33]. Through the use of semi-quantitative RT-PCR (Figure 1A) and Western blot analysis (Figure 1B), we examined the CK2α gene and protein in eight of NSCLC cell lines. Data showed that CK2α is expressed in all these cancer cells. Among them, at least five cell lines (A549, A427, H1299, H358 and H838) showed relatively higher expression of CK2α both at the mRNA and protein levels. CK2α expression was previously shown to be minimal in normal lung cells [34]. Gli1 gene and protein expressions were broadly detected in all cell lines except H358. Interestingly, there appeared to be a correlation between the expression of CK2α and Gli1 in these cell lines. We performed real-time RT-PCR of CK2α and Gli1 in 100 primary NSCLC samples. A mild correlation between CK2α and Gli1 mRNA levels was found in these tissues (r = 0.37, *P*<0.05) (Figure 1C). For subsequent experimental studies, A549 was chosen because the status of cancer-related pathways in A549 cells has been well characterized. H1299 was also chosen because of its relatively higher expression of CK2α and Gli1 genes.

### CK2α Knockdown Inhibits Hh/Gli Signaling Through Down-Regulating Gli1

**Figure 2:**
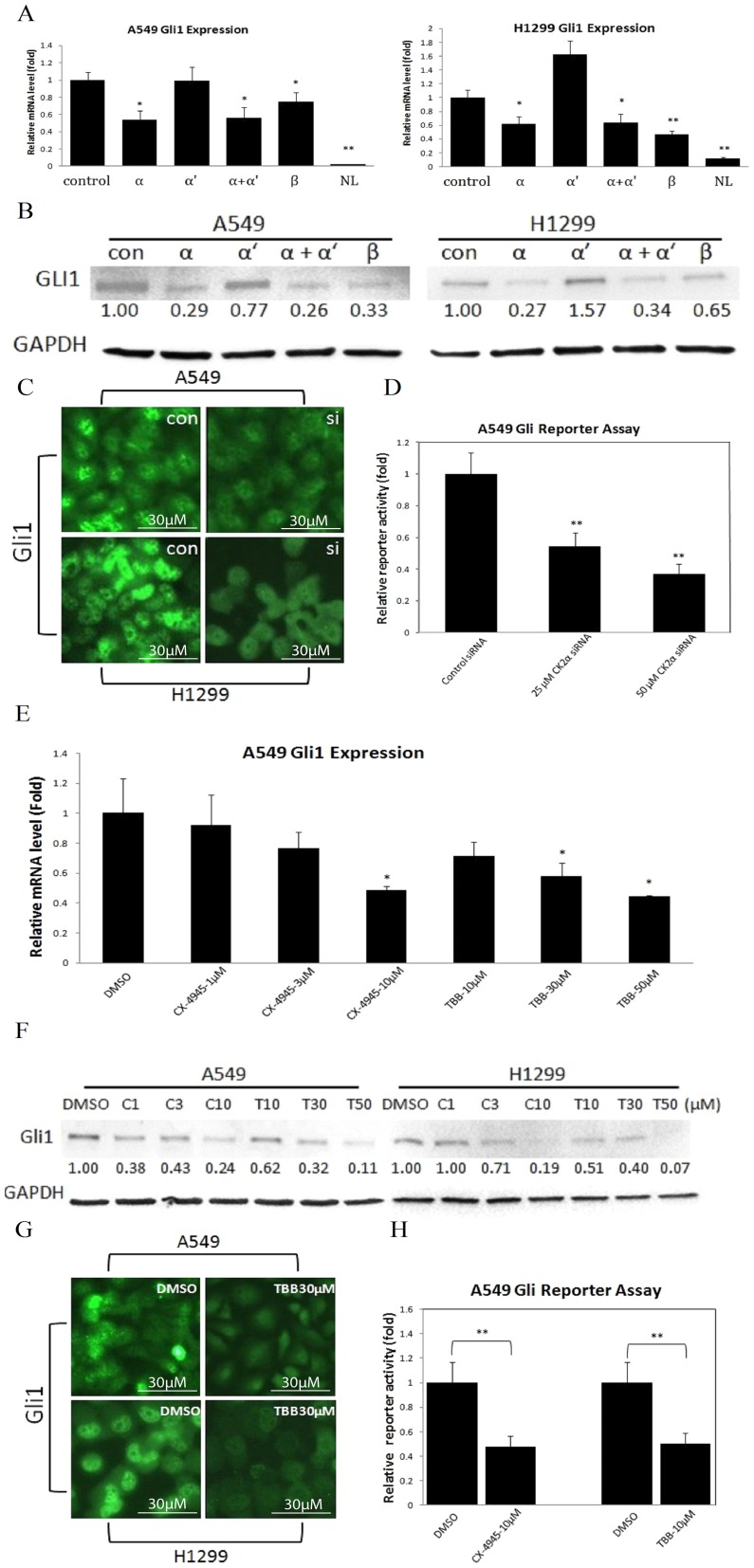
CK2α Inhibition Down-Regulates Gli1 Expression and Transcriptional Activity. (A) Quantitative Gli1 mRNA levels after treatment with CK2 subunit-specific siRNA detected by real-time RT-PCR. Silencing of CK2α significantly reduced Gli1 mRNA levels both in A549 and H1299 cell lines (by 50% and 45%, respectively). Silencing of CK2β also resulted in a significant decrease of Gli1 in both cell lines. Minimal Gli1 mRNA level was noticed in the normal lung (NL). * *P*<0.05, ** *P*<0.01, Student’s t-test. (B) Protein levels detected by Western blot. Silencing of CK2α led to 71% (A549) and 73% (H1299) reduction of Gli1, while silencing of CK2β led to 67% (A549) and 35% (H1299) decrease of Gli1. Treatment by CK2α’-targeting siRNA produced no obvious difference, rather than decrease of Gli1 in A549, and produced a 60% increase in H1299, both in mRNA and protein levels. (C) Localization of Gli1 detected by green fluorescence. The Gli1 protein mainly localizes in the nuclear compartment of the cells, and shows a great reduction after CK2α knockdown. Scale bar = 30 µm. (D) The transcriptional activity of Hh pathway in A549 detected by Gli reporter assay. Silencing of CK2α resulted in a significant decrease (more than 40% at 25 µM and 60% at 50 µM) of the transcriptional activity, compared with the control siRNA. * *P*<0.05, ** *P*<0.01, Student’s t-test. (E) Quantitative Gli1 mRNA levels after treatment with TBB and CX-4945 by real-time RT-PCR. Gli1 expression in A549 decreased noticeably at the dosage levels of 1 µM CX-4945 and 10 µM TBB, significantly at 10 µM CX-4945 and 50 µM TBB (50% and 60%). * *P*<0.05, ** *P*<0.01, Student’s t-test. (F) Protein levels detected by Western blot. In protein level, these decreases rose to 76% (10 µM CX-4945) and 89% (50 µM TBB) in A549, and 81% (10 µM CX-4945) and 93% (50 µM TBB) in H1299, respectively. (G) Protein expression of Gli1 detected by green fluorescence. Cells were treat with 30 µM TBB or vehicle DMSO, immunoflorescence staining with anti-Gli1 mAb on cultured cells showed a distinctly lower green florescence in the presence of 30 µM TBB. (Scale bar = 30 µm). (H) The transcriptional activity of Hh pathway in A549 detected by Gli reporter assay. A 50% decrease was detected in the presence of 10 µM CX-4945 or 10 µM TBB. * *P*<0.05, ** *P*<0.01, Student’s t-test.

To investigate whether CK2 suppression have an effect on the Hh pathway, we silenced CK2 expression using CK2 subunit-specific siRNAs. Forty-eight hours after transfection, the efficiency of RNA interference was monitored by semi-quantitative RT-PCR (Figure S1). The corresponding mRNA levels of the three subunits decreased, and the knockdown of α and α′ was confirmed by co-transfection of their siRNAs. The expression of the indicated Hh pathway components was also determined (Figure S2). Overall, the RT-PCR results showed that Ptc1 and Gli1 mRNA levels in A549 and H1299 cells were consistently down-regulated after CK2α or CK2β knockdown, whereas minimal changes were observed in other Hh pathway components. By real-time RT-PCR, we confirmed that the silencing of CK2α significantly inhibited Gli1 expression both in A549 and H1299 cell lines (Figure 2A). Silencing of CK2β also resulted in a significant decrease of Gli1 in both cell lines (Figure 2A). In addition, Gli1 expression was minimal in the normal lung control. At the protein level, silencing of CK2α led to a 71% (A549) and a 73% (H1299) decrease of Gli1, while silencing of CK2β led to a 67% (A549) and a 35% (H1299) decrease of Gli1. Treatment with CK2α′-targeting siRNA produced no obvious difference, rather than decrease of Gli1 in A549, and produced a 60% increase in H1299, both at the mRNA and protein levels (Figure 2B). Furthermore, we performed immunofluorescence staining with a monoclonal anti-Gli1 antibody, since nuclear localization of Gli1 reflects activity of the Hh pathway [35]. Nuclear Gli1 proteins were dramatically decreased in the presence of CK2α siRNA (Figure 2C). Moreover, silencing of CK2α resulted in a significant decrease (45% at 25 µM and 60% at 50 µM, P<0.01) in the Gli1-boosted Gli reporter activity, compared with the non-targeting siRNA (control) (Figure 2D).

### Small-Molecule CK2α Inhibitors Down-Regulate Gli1 Expression and Transcriptional Activity

To further validate the role played by CK2α in the Hh pathway, we used two small-molecule CK2α inhibitors: TBB (4,5,6,7-tetrabromobenzotriazole), a well-known inhibitor of CK2α [36], and CX-4945 (5-(3-chlorophenylamino)benzo[c] [2], [6]naphthyridine-8-carboxylic acid), a first-in-class, selective, oral inhibitor of CK2α under investigation in Phase 1 clinical trials [37].

Cells were treated with various concentrations of TBB (10, 30, 50 µM) or CX-4945 (1, 3, 10 µM), or with the vehicle DMSO for 48 hours. Treatments with TBB or CX-4945 led to a dose-dependent decrease of Gli1 mRNA and protein levels both in A549 and H1299 cell lines (Figure 2E, 2F and S3). The quantitative mRNA levels were detected by real-time RT-PCR. As shown in Figure 2E, Gli1 expression in A549 decreased noticeably at the dosage levels of 1 µM CX-4945 and 10 µM TBB, and decreased significantly at 10 µM CX-4945 and 50 µM TBB (50% and 60%, *P*<0.05). At the protein level, these decreases reached to 76% (10 µM CX-4945) and 89% (50 µM TBB) in A549, and 81% (10 µM CX-4945) and 93% (50 µM TBB) in H1299, respectively (Figure 2F). Immunofluorescence staining with a monoclonal anti-Gli1 antibody on cultured cells showed a dramatically lower green florescence in the presence of 30 µM TBB (Figure 2G). We then demonstrated that the small molecules inhibited Gli reporter activity in A549 cell line, where a significant decrease (55%, P<0.01) was detected in the presence of 10 µM CX-4945 or 10 µM TBB (Figure 2H). These results are consistent with what we found in siRNA studies.

### Forced Over-Expression of the CK2α Gene Leads to Gli1 Up-Regulation and Transcriptional Activation

**Figure 3:**
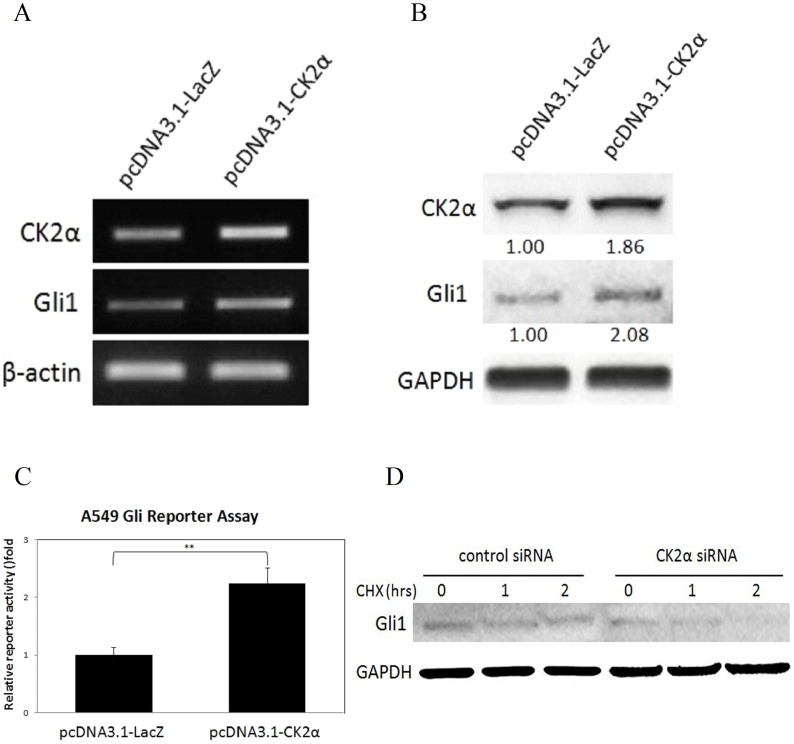
Over-Expression of CK2α Gene Leads to Gli1 Up-Regulation and Transcriptional Activation, and Loss of CK2α Function Promotes Gli1 Degradation. (A)A549 cells were transfected either with a pcDNA3.1-CK2α or control pcDNA3.1-LacZ plasmid vectors, the over-expressed CK2α and up-regulated Gli1was detected by real-time RT-PCR. * *P*<0.05, ** *P*<0.01, Student’s t-test. (B)Western blot. The protein level of Gli1 was up-regulated in this system with a two-fold increase. (C)Moreover, the reporter assay also showed a significant (more than 2-fold) increase of the Gli1 transcriptional activity. * *P*<0.05, ** *P*<0.01, Student’s t-test. (D)In a protein degradation analysis, A549 cells showed reduced Gli1 protein at the time point of 2 hours after treated with CK2α siRNA.

To confirm whether the CK2 gene positively affects the transcriptional activity of Gli1, we transfected A549 cells with either a pcDNA3.1-CK2α or control pcDNA3.1-LacZ plasmid. As expected, the over-expression of CK2α was attributed to the activation of Gli1 in the A549 cell line. The over-expression of CK2α was detected by RT-PCR (Figure 3A) and Western blot (Figure 3B). The elevated Gli1 mRNA level was also detected by RT-PCR (Figure 3A). By Western blot analysis, we showed the elevated Gli1 protein level (2.35 folds) in cells with ectopic over-expression of CK2α (Figure 3B). Moreover, the reporter assay also showed a significant (>2 folds, P<0.01) increase of Gli1 transcriptional activity (Figure 3C). These findings suggest that the over-expression of CK2α gene leads to an up-regulation of Gli1 gene expression and transcriptional activity.

### The Silencing of CK2α Promotes Gli1 Degradation

We further carried out a time-course experiment to examine Gli1 half-time. A549 cells were transfected with CK2α or control siRNA, and Gli1 protein levels were detected at the time points of 0, 1 and 2 hours after treatment with the protein inhibitor, cycloheximide. In the CK2α knockdown group, the Gli1 protein level reduced to mimimum at 2 hours after treatment with cycloheximide (when compared with that at 0 hour), which suggested that the half life of Gli1 after CK2α siRNA knockdown is <2 hours. These data indicate that CK2α knockdown results in degradation of Gli1 (Figure 3D). This, in turn, suggests that CK2α regulates Gli1 activity by preventing its degradation.

### CK2α Knockdown Down-Regulates Hh Downstream Genes and Reduces SP Profile via ABCG2 in A549

**Figure 4:**
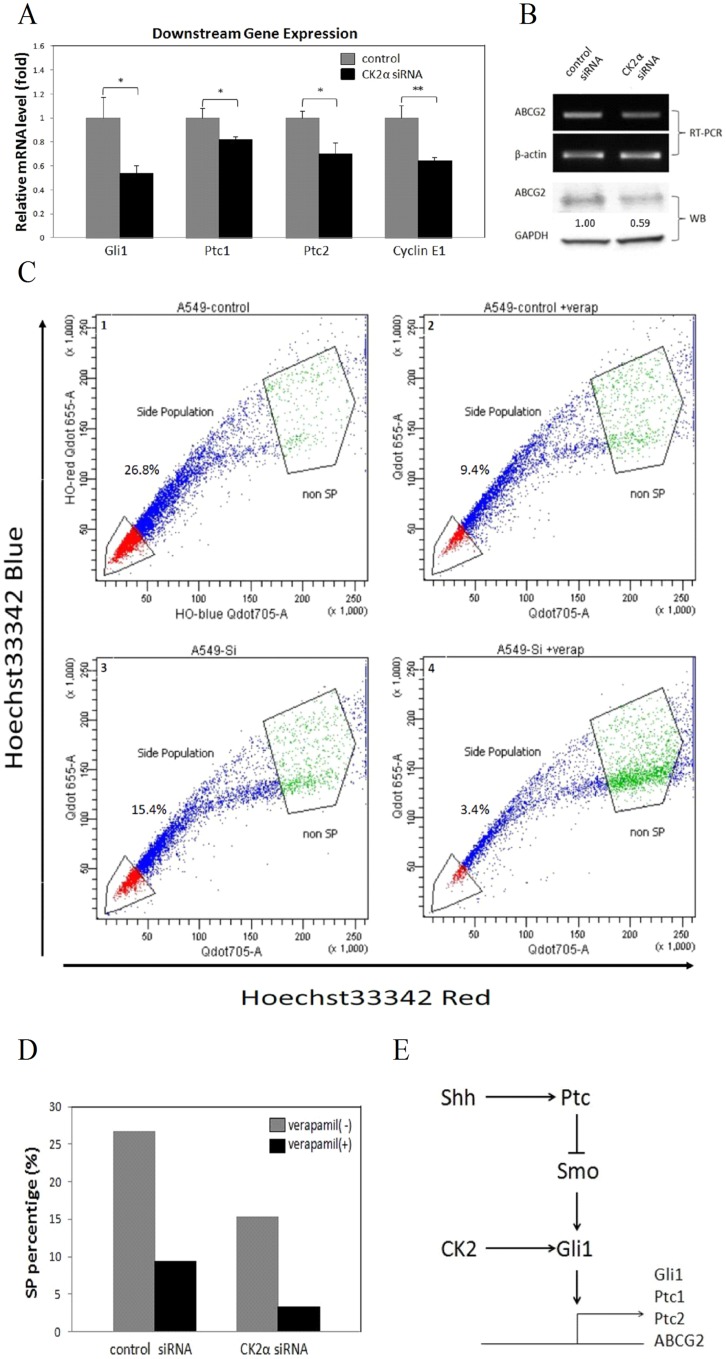
CK2α Knockdown Down-Regulates Hh Signal Pathway Transduction and Reduces SP Proportion in A549. (A) Hh downstream genes expression detected by real-time RT-PCR. The mRNA level of the four Hh target genes (Gli1, Ptc1, Ptc2 and Cyclin E1) decreased significantly after CK2α knockdown. * *P*<0.05, ** *P*<0.01, Student’s t-test. (B) The expression of ABCG2 decreased in CK2α-silenced A549 cells. (C) The proportion of SP cells dropped from 26.8% to 15.4%, and from 9.4% to 3.4% in presence of 50 µM verapamil, respectively. Cells were labeled with the Hoechst 33342 and then analyzed by flow cytometry. (right) Results when the cells were treated with 50 µM verapamil during the labeling procedure. The SP is outlined and shown as a percentage of the total cell population. These experiments were repeated at three times with similar results. (D) Bar chart for (C). (E) Model of Hh/Gli1 signaling regulated by CK2. Details are described in the text.

Hh reportedly controls the proliferation of several cell types through various molecular mechanisms and targets downstream genes, including Ptc, Cyclin family members, and self-induction of Gli1 [20], [38]. We analyzed mRNA levels of four (Gli1, Ptc1, Ptc2 and Cyclin E1) of these genes by using real-time RT-PCR (Figure 4A). The expression of the four Hh target genes decreased significantly (*P*<0.05) after CK2α knockdown, which suggests a depressed transcriptional activity of the Hh pathway.

It has been demonstrated that ABCG2 is the primary contributor to the SP phenotype in several cancer cell lines including A549 [28]. The SP phenotype has been characterized in a series of studies of cancer stem cells derived from prostate, breast, colon, glioma, bladder, ovary, cervix, and melanoma [29], [39], [40], [41], [42]. Recent studies show that ABCG2 is a direct target of the Hh pathway which is involved in stem cell maintenance [27]. We first examined ABCG2 expression after CK2α knockdown and found that the ABCG2 level decreased dramatically in CK2α-silenced A549 cells (Figure 4B). Consistent with other findings, relatively higher percentage (26.8%) of A549 cells were classified as SP cells in our study. In the presence of verapamil, an ABC transporter inhibitor, the proportion of SP cells dropped to 9.4%. After treatment with CK2α siRNA, the proportion dropped to 15.4% (without verapamil) or 3.4% (with verapamil) (Figure 4C and 4D). We then sorted SP and non-SP cells in this system. In further analysis by semi-quantitative RT-PCR, the sorted SP cells showed higher expression of ABCG2 than did non-SP cells, but no difference of ABCG2 expression in SP or non-SP cells was shown between the CK2α siRNA and control (Figure S4). In brief, we showed a 42.5% reduction of SP proportion after CK2α knockdown.

## Discussion

Our results suggest that CK2 is a positive regulator in Hh/Gli1 signaling in human lung cancer. This is supported by several lines of evidence. First of all, the correlation of CK2 and Gli1 expressions were noticed in several lung cancer cell lines. We further demonstrated a linear correlation in 100 primary NSCLC tissues. Secondly, the inhibition of CK2α by siRNA or small-molecular inhibitors resulted in down-regulation of Gli1 expression and transcriptional activity. Thirdly, forced over-expression of CK2α resulted in increased Gli1 expression and transcriptional activity. Finally, CK2α knockdown led to a reduction of side population via down-regulating ABCG2, a direct target of Hh/Gli signaling [27].

To date, there is no evidence for the correlation between CK2 and Hh/Gli signaling in human cancer cells, although CK2 was suggested as a positive regulator of the Hh signal transduction pathway and two serine residues in Smo were phosphorylated by CK2 in *Drosophila* [43]. However, this mechanism on Smo phosphorylation by CK2 does not appear to apply to humans, as these two Smo residues are not conserved in human Smo when aligned with Clustal W [44] (Figure S5). Furthermore, several studies have suggested that mammalian Smo and *Drosophila* Smo are regulated by fundamentally distinct mechanisms [45], [46]. Recently, Chen et al. demonstrated that mammalian Smo is activated through multi-site phosphorylation by CK1α and GRK2 and proposed two-step mechanism for Smo phosphorylation in mammalian cells [47].

To investigate the potential mechanism through which CK2 positively regulates human Gli1 expression and transcriptional activity, we performed protein degradation assay of Gli1 after treatment with CK2α siRNA. Our results indicated that CK2 silencing reduces the half-life of human Gil1 protein in A549 cells. Furthermore, both CK2 siRNA and small molecule inhibitors down-regulated Gli1-boosted transcriptional activity. Moreover, forced over-expression of CK2α resulted in Gli1 transcriptional activity. In addition, we found two predicted CK2 phosphorylation sites in human Gli1 by using Scansite with medium stringency [48] (Figure S6). Our data suggest that CK2 may regulate Gli1 in human cancer cells in a similar manner with that in *Drosophila*. For instance, Jia et al. reported that CK2 directly phosphorylates Ci in *Drosophila*, and then prevents its ubiquitination and degradation [43]. On the other hand, it has been proposed that glycogen synthase kinase-3 beta negatively regulates Gli1 transcription factors cooperating with other kinases such as PKA and CK1s in lung cancer A549 cells [49]. Thus, two steps are probably involved in the positive regulation of Gli1 by CK2. In the first step, inhibition of CK2 promotes Gli1 degradation, followed by reduced accumulation of Gli1 in nuclear compartment. In the second step, as the key transcription factor of the Hh pathway, the reduced Gli1 protein subsequently suppresses the transcription of the Hh target genes. This in turn, forms a feedback loop to further decrease the expression level of Gli1, which is acting as a target gene of the pathway (Figure 4E). Although the mechanism of CK2 regulation in human cancers remains largely unknown, there is evidence that CK2 is essential for Wnt/beta-catenin signaling [50], [51]. For instance, it was implicated that CK2 may bind and phosphorylate β-catenin and promotes its degradation [52]. Taken together, these results suggest that CK2α may stabilize of human Gli1 protein through direct phosphorylation. Further studies are needed to elucidate the precise mechanisms.

The Hh pathway transcription factor Gli1 regulates the expression of the ABC transporter protein ABCG2 by directly binding to the ABCG2 promoter [27], ABCG2 is a molecular determinant of the SP phenotype and expressed at higher levels in SP cells, compared with non-SP cells [28], [31]. The SP cells are reportedly enriched with cancer stem cells, as they shows stem-cell properties (highly tumurigenic and chemo-resistant). Previous studies have detected SP phenotype with higher ABCG2 expression level and implicated ABCG2 as a CSC marker in lung cancer A549 cells [28]. For instance, SP cells in A549 showed more tumurigenic potential [29]. In our study, the target gene of Hh/Gli signaling ABCG2 was down-regulated after CK2α inhibition, where ABCG2-driven side population was reduced subsequently. Thus, we report that CK2 participates in CSCs maintenance by regulating Hh/Gli signaling.

Hh pathway may play key roles in the maintenance of CSCs, however the druggable targets in Hh pathway is very limited. CK2 provide an additional target for the inhibition of Hh/Gli signaling. CK2 inhibitors have not been extensively developed as therapeutic agents, partially due to that the ATP-binding pocket of CK2 is not as druggable as some other protein kinases [50], [53], [54] . To date, only one small-molecule CK2 inhibitor has been entered to clinical trials as a potential anticancer drug. CX-4945, a highly selective CK2 small molecule inhibitor, is a promising first-in-class oral therapeutic agent targeting multiple human cancers. CX-4945 shows a favorable safety profile in Phase I clinical trials [55]. In addition, CIGB-300 (a synthetic peptide-based drug targeting the CK2 phosphoaceptor domain) has proved to be safe and of clinical benefit in Phase I cervical cancer trials [56].

In summary, we report that CK2 is a positive regulator in the Hh/Gli signaling pathway, and the inhibition of CK2α down-regulates Hh/Gli signaling in human lung cancer cells. Given the emerging importance of Hh/Gli signaling in tumor initiation and progression, our findings provide an important evidence for the potential benefits of CK2 inhibitors.

## Materials and Methods

### Cell Culture and Small Molecule Treatment

Human NSCLC cell lines (A549, A427, H460, H1299, H1650, H358, H838 and H322) were obtained from American Type Culture Collections (Manassas, VA). Cells were routinely maintained in RPMI-1640 supplemented with 10% heat-inactivated fetal bovine serum, penicillin (100 µg/ml) and streptomycin (100 µg/ml). All cells were routinely cultivated at 37°C in a humid incubator with 5% CO2. Treatment with CX-4945 (Synkinase, San Diego, CA) and TBB (Sigma, St. Louis, MO) dissolved in DMSO was administered at several dosages (1, 3 and 10 µM of CX4945; 10, 30, 50 µM of TBB). Cells were grown in medium for 48 hours after treatment.

### SiRNA and Plasmid DNA Transfection

CK2α, CK2α′ and CK2β-specific siRNAs (ON-TARGET *plus* SMARTpool) and control RNA were purchased from Thermo Scientific (Waltham, MA). In brief, cells were seeded in a 6-well plate as 10^5^ cells/well one day before transfection, with a target of 30–50% confluency at the time of transfection. Cells were transfected with 50 nmol/L of siRNA using Lipofectamine RNAiMAX (Invitrogen, Carlsbad, CA) according to the manufacturer’s protocol. Adequate inhibition of the siRNA-mediated knockdown was confirmed by RT-PCR. The pcDNA3.1-CK2α or control pcDNA3.1-LacZ plasmid vectors were then transfected into the A549 cells (0.5 µg/ml in 24-well plate) using Lipofectamine 2000 transfection reagent (Invitrogen), according to the manufacturer’s protocol. Cells were harvested for RT-PCR and Western blot or used in reporter assays at 48 hours post-transfection.

### RNA Isolation, cDNA Synthesis and Semi-quantitative RT-PCR

Isolation of RNA was performed using RNeasy Mini kit (Qiagen, Valencia, CA). Human Lung Total RNA was purchased from Applied Biosystems (Foster City, CA). Five-hundred ng of total RNA was converted into 20 µl cDNA using iScript cDNA Synthesis Kits (Bio-Rad, Hercules, CA,) according to the manufacturer’s recommendations. PCR bands were visualized under UV light and photographed.

### Real-Time RT-PCR

A total of 2 µl of the reverse transcription reaction were used as template for real-time detection of Gli1 expression using TaqMan Technology on an Applied Biosystems 7000 sequence detection system (Applied Biosystems, Foster City, CA). Gene expression was quantitated for the tested genes (Gli1, Ptc1, Ptc2 and Cyclin E1) and endogenous control gene b-glucuronidase (GUSB) using the primer and probe sequences commercially (Applied Biosystems).

### Western Blot Analysis and Immunofluorescence Staining

Whole protein was extracted by M-PER Mammalian Protein Extraction Reagent (Thermo) from cell lines added with Phosphatase Inhibitor Cocktail Set II (Calbiochem, San Diego, CA) and Complete Protease Inhibitor Cocktails (Roche, Lewes, UK) according to manufactures’ protocols. The proteins were separated on 4–15% gradient SDS–polyacrylamide gels and transferred to Immobilon-P membranes (Millipore, Bellerica, MA). The following primary antibodies were used: anti-CK2α (Millipore), anti-Gli1 (Cell Signaling, Beverly, MA), anti-ABCG2 (Millipore), and anti-GAPDH (Trevigen, Gaithersburg, MD). After being incubated with appropriate secondary antibodies, the antigen-antibody complexes were detected by using an ECL blotting analysis system (Amersham Pharmacia Biotech, Piscataway, NJ). Immunofluorescence staining was carried out. Examination was done using laser scanning confocal microscopy (LSM510, Carl Zeiss, Oakland, CA). Digital images of single confocal slices were prepared using Adobe Photoshop 6.0.

### Protein Degradation Assay

The CK2α- and control siRNA-transtected A549 cells were exposed to 50 µg/ml cycloheximide and harvested at the time points of 0 and 6 hours. Total cellular proteins were extracted and were analyzed by western blot analysis.

### Luciferase Reporter Assays

To measure Gli-mediated Hh transcriptional activity, the luciferase reporter constructs, 8× wild-type Gli binding site (8× Gli^wt^ Luc) or 8× mutant Gli binding site (8× Gli^mut^ Luc) plasmids [57] and a human Gli1 expression vector (pcDNA3.1-Gli1) were co-transfected into A549 cells in 24-well plate. The *Renilla* luciferase pRL-TK plasmid (Promega, Madison, WI), whose expression is driven by the housekeeping thymidine kinase gene promoter, was co-transfected to normalize for transfection efficiency. All transfection experiments were performed using the Lipofectamine2000 (Invitrogen) in accordance with the manufacturer’s instructions. After 24 h cells were lysed and luciferase assays were performed as described previously [58]. Results are expressed as fold induction, which is the ratio of luciferase activity induced in Gli-transfected cells relative to basal luciferase activity in control transfected A549 cells. All experiments were performed in triplicate; means and standard errors were calculated using Student’s *t*-test.

### Flow Cytometry Analysis and Sorting

Identification of SP cells was performed as described previously by Goodell et al. [42]. The A549 cells were incubated with 5 µg/ml Hoechst 33342 dye (Invitrogen) for 90 min with and without 50 µM verapamil. Cell samples were analyzed and sorted using a Moflo MLS cell sorter (Beckman-Coulter, Hialeah, FL) with UV capabilities and SUMMIT software for data acquisition and analysis. An argon laser was used to excite the Hoechst dye. Fluorescence emission was collected with a 405/30 nm bandpass filter for Hoechst blue and a 670/40 nm bandpass filter for Hoechst red. Dead cells were excluded by propidium iodide fluorescence at 670/30 nm. The SP and non-SP cells were sorted for further test of their ABCG2 gene expression.

### Statistical Analysis

Data were expressed as mean ± standard deviation (SD) from three independent experiments. All of the statistical analyses were performed using the SPSS 13.0 for Windows software system (SPSS Inc, Chicago, IL). Student’s *t*-test was used to compare the differences among groups. Pearson product correlation tests (in the form of a correlation matrix) were used to analyze the mRNA level of CK2 and Gli1. A significant difference was declared if the *P* value from a two-tailed test was less than 0.05 (* *P*<0.05, ** *P*<0.01).

Supplementary materials and methods are shown in Text S1.

## Supporting Information

Figure S1 **Silencing of CK2 substrates genes expression by siRNA. Forty-eight hours after transfection, the efficiency of RNA interference was monitored by semi-quantitative RT-PCR.** The corresponding mRNA levels of the three subunits decreased, and the knockdown of α and α′ was confirmed by co-transfection.
(TIF)

Figure S2 **The expression of the indicated Hh pathway components, detected by semi-quantitative RT-PCR.** The results showed that Ptc and Gli1 gene expression in A549 and H1299 was consistently down-regulated after CK2α and β knockdown, whereas no obvious changes were revealed in other HH pathway components.
(TIF)

Figure S3 **Treatments with TBB or CX4945 led to a dose-dependent decrease of Gli1 mRNA level both in A549 and H1299, which was detected by semi-quantitative RT-PCR.**
(TIF)

Figure S4 **The sorted SP cells showed higher expression of ABCG2 than non-SP cells in semi-quantitative RT-PCR, however, no difference of ABCG2 expression in SP or non-SP cells was shown between the CK2α siRNA and control.**
(TIF)

Figure S5 **Multiple sequence alignment of Smoothened with peptides indentified by Proteomics analysis.** Hh membrane receptor Smo in human (NCBI Reference Sequence: EAL24102.1) and Drosophila melanogaster (NCBI Reference Sequence: NP_523443.1) was aligned with Clustal W software. The two serine phosphorylation sites of CK2 (indicated in box) in Drosophila do not exist in humans, indicating that the regulation of Hh by CK2 is a Smo phosphorylation-independent reaction.
(TIF)

Figure S6 **The phosphorylation prediction results of human Gli1 with CK2. Two CK2 phosphorylation sites (indicated in box) in Gli1 (NCBI Reference Sequence: AAM13391.1) were predicted using Scansite 2.0 with medium stringency.**
(TIF)

Text S1 Supplementary materials and methods.
(DOC)
