# Supplementary figures and images for: Inhibition of CK2α Down-Regulates Hedgehog/Gli Signaling Leading to a Reduction of a Stem-Like Side Population in Human Lung Cancer Cells
Source: PLoS One. 2012 Jun 29;7(6):e38996. doi: 10.1371/journal.pone.0038996 (PMC3387212; doi:10.1371/journal.pone.0038996)

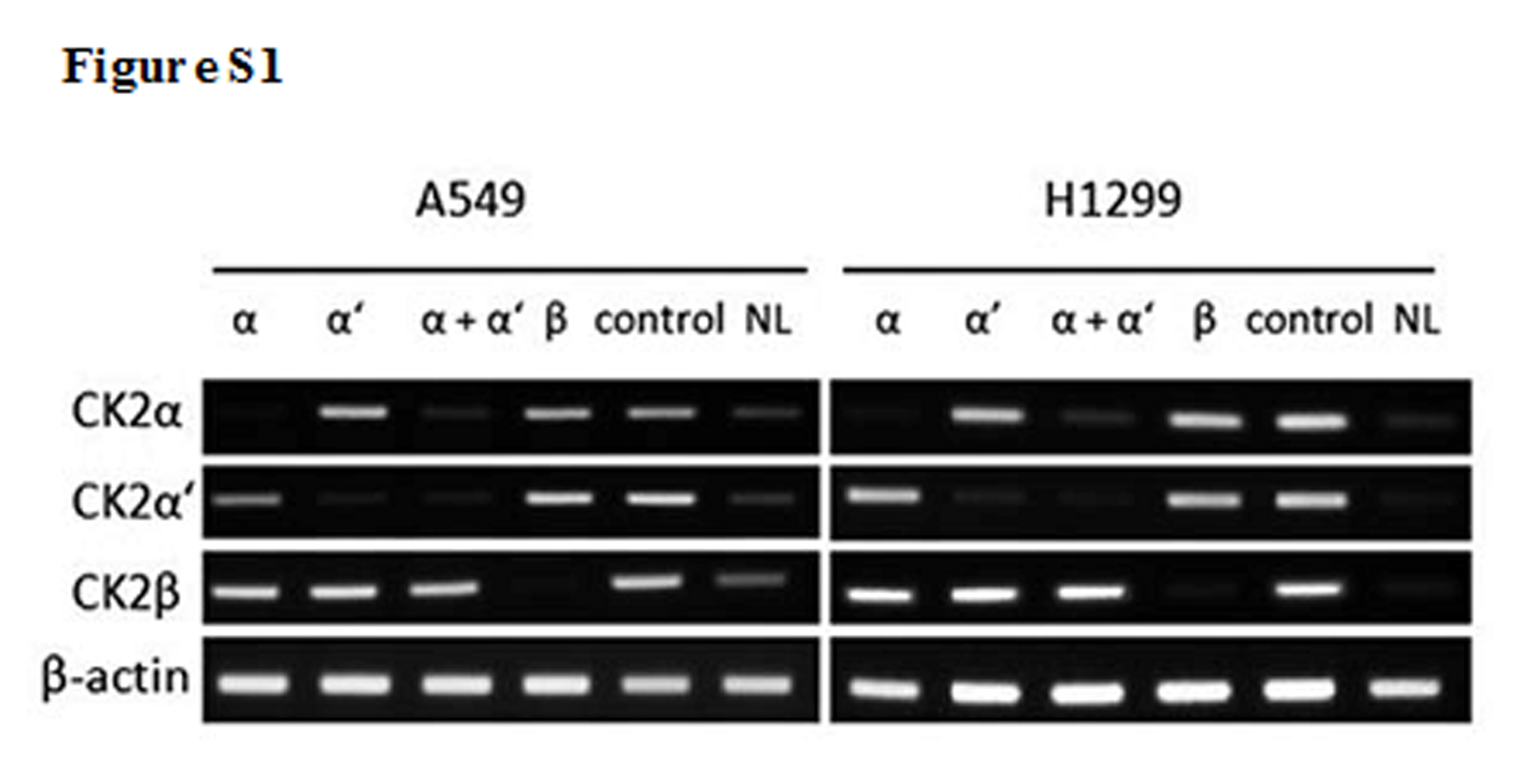

Supplement: Figure S1 — Silencing of CK2 substrates genes expression by siRNA. Forty-eight hours after transfection, the efficiency of RNA interference was monitored by semi-quantitative RT-PCR. The corresponding mRNA levels of the three subunits decreased, and the knockdown of α and α′ was confirmed by co-transfection. (TIF) [file pone.0038996.s001.tif]

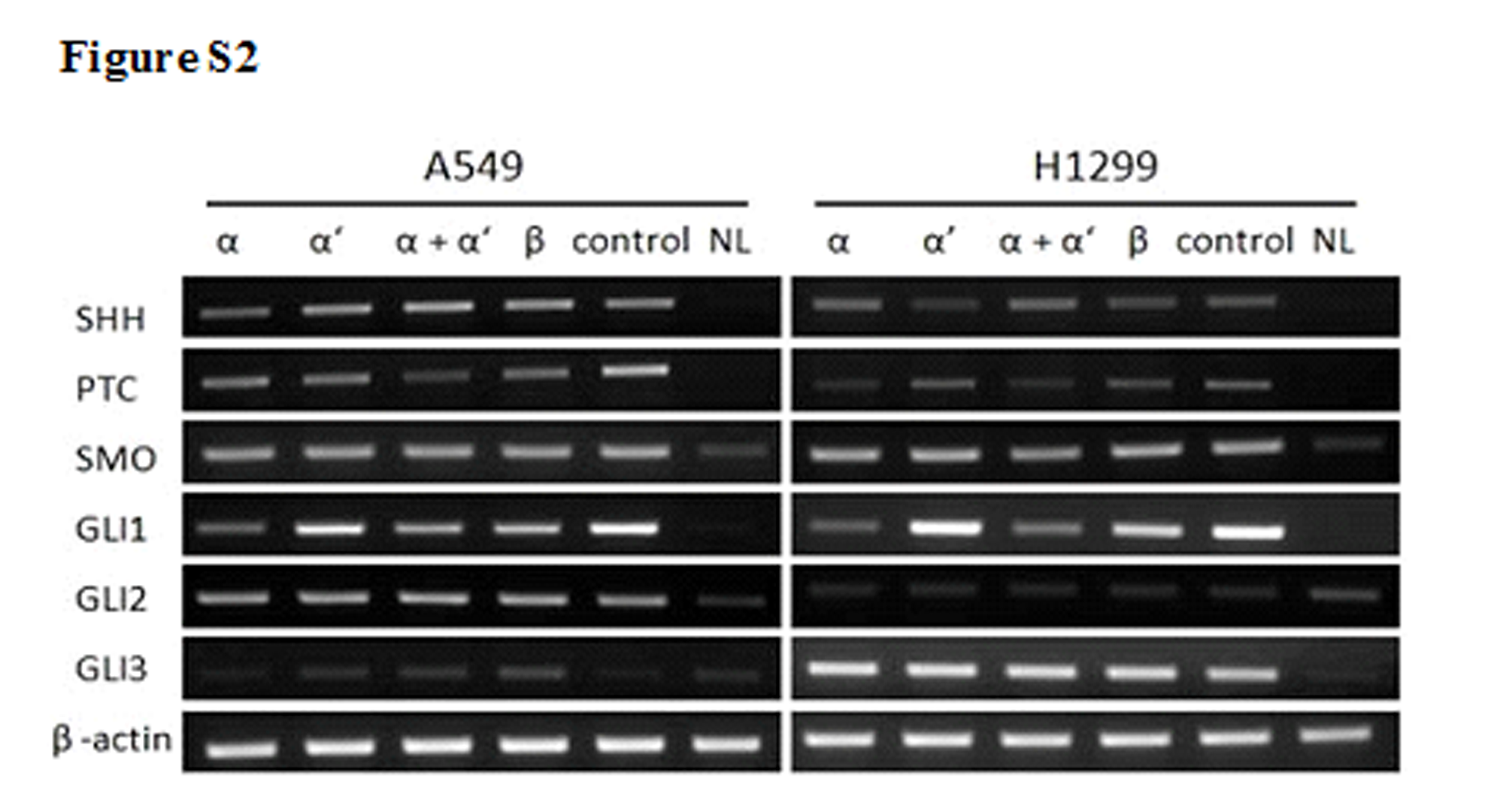

Supplement: Figure S2 — The expression of the indicated Hh pathway components, detected by semi-quantitative RT-PCR. The results showed that Ptc and Gli1 gene expression in A549 and H1299 was consistently down-regulated after CK2α and β knockdown, whereas no obvious changes were revealed in other HH pathway components. (TIF) [file pone.0038996.s002.tif]

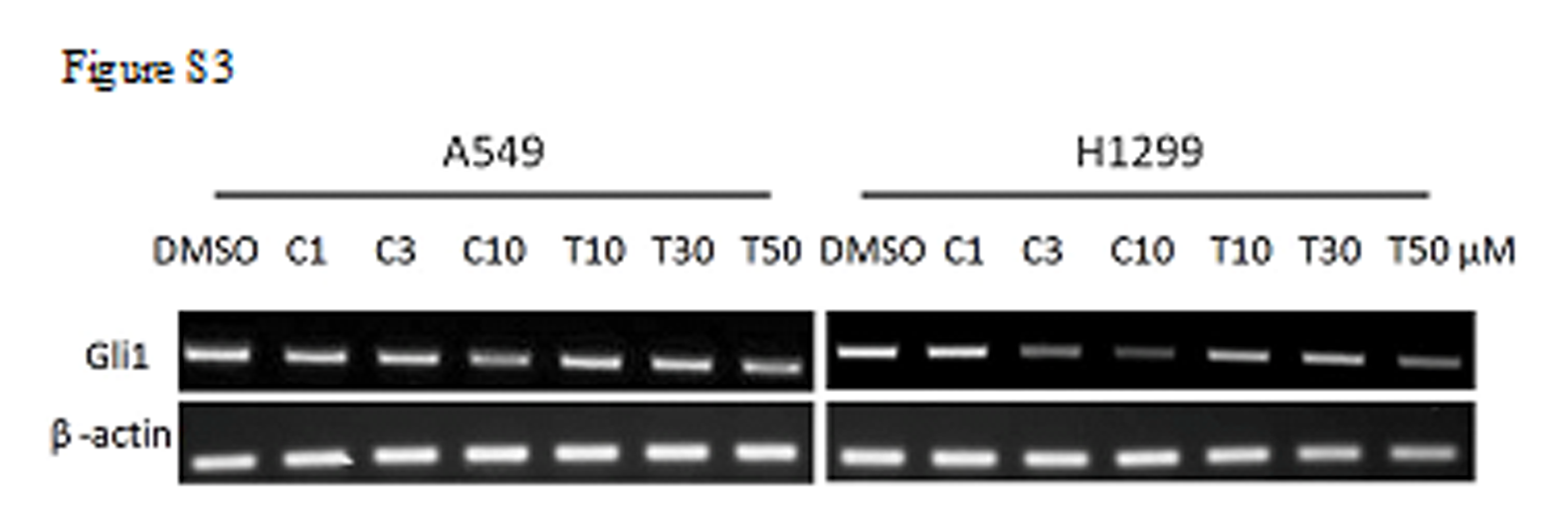

Supplement: Figure S3 — Treatments with TBB or CX4945 led to a dose-dependent decrease of Gli1 mRNA level both in A549 and H1299, which was detected by semi-quantitative RT-PCR. (TIF) [file pone.0038996.s003.tif]

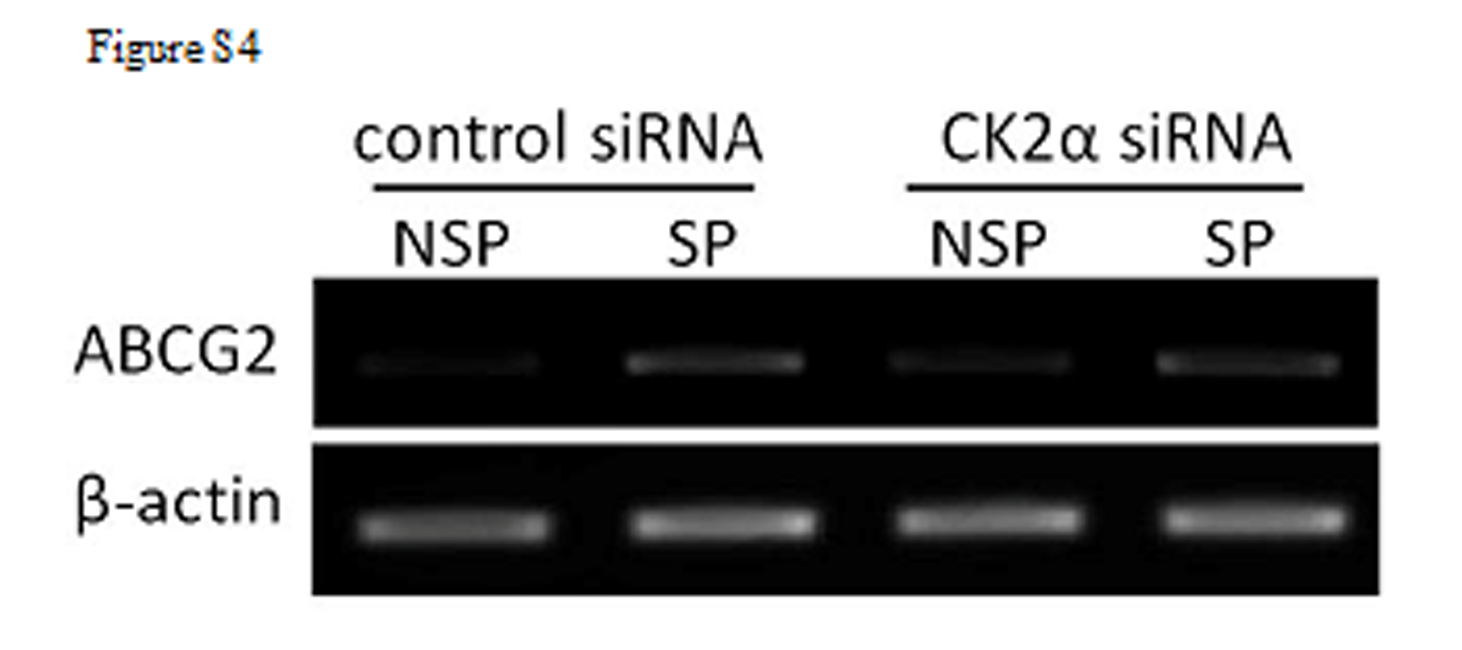

Supplement: Figure S4 — The sorted SP cells showed higher expression of ABCG2 than non-SP cells in semi-quantitative RT-PCR, however, no difference of ABCG2 expression in SP or non-SP cells was shown between the CK2α siRNA and control. (TIF) [file pone.0038996.s004.tif]

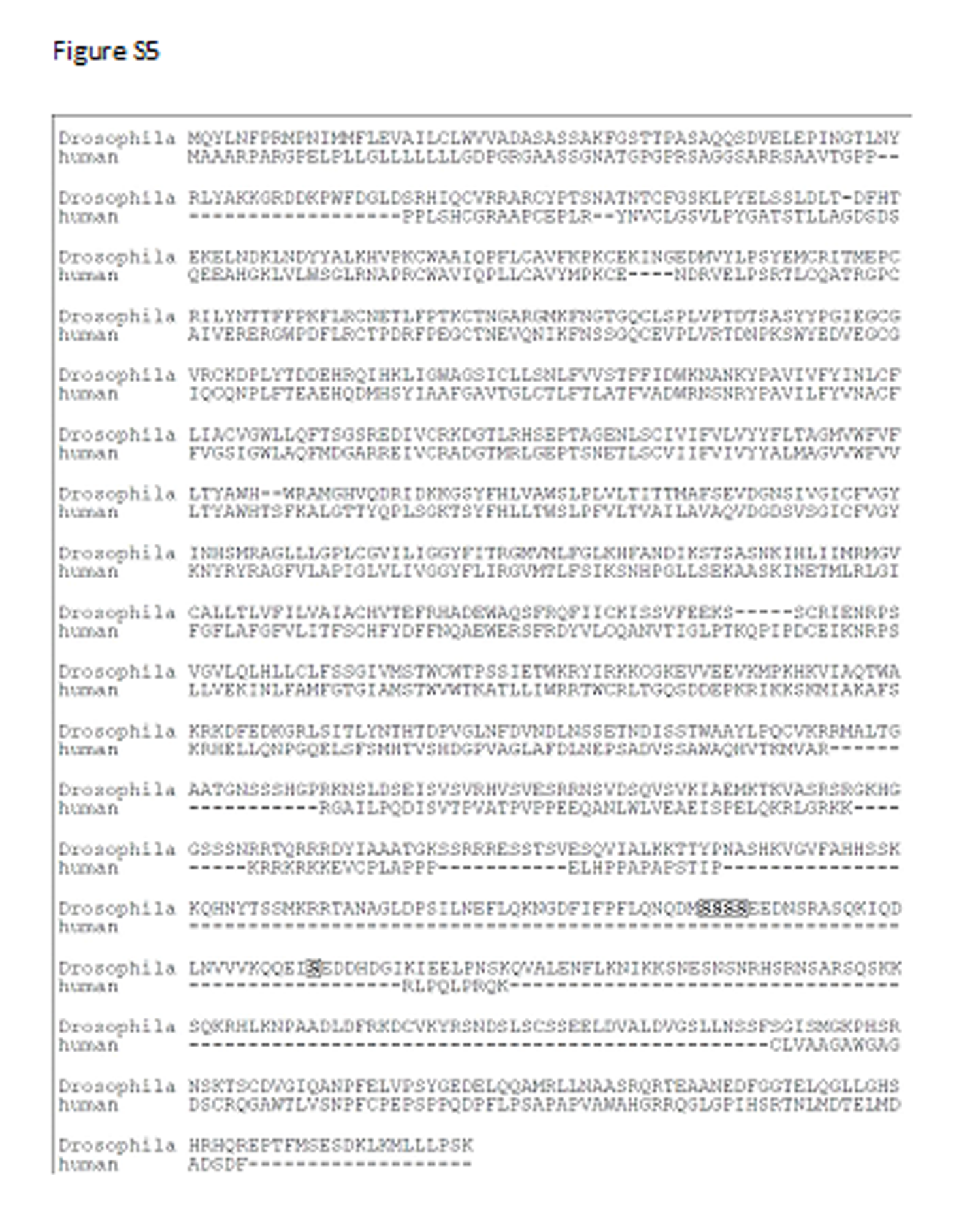

Supplement: Figure S5 — Multiple sequence alignment of Smoothened with peptides indentified by Proteomics analysis. Hh membrane receptor Smo in human (NCBI Reference Sequence: EAL24102.1) and Drosophila melanogaster (NCBI Reference Sequence: NP_523443.1) was aligned with Clustal W software. The two serine phosphorylation sites of CK2 (indicated in box) in Drosophila do not exist in humans, indicating that the regulation of Hh by CK2 is a Smo phosphorylation-independent reaction. (TIF) [file pone.0038996.s005.tif]

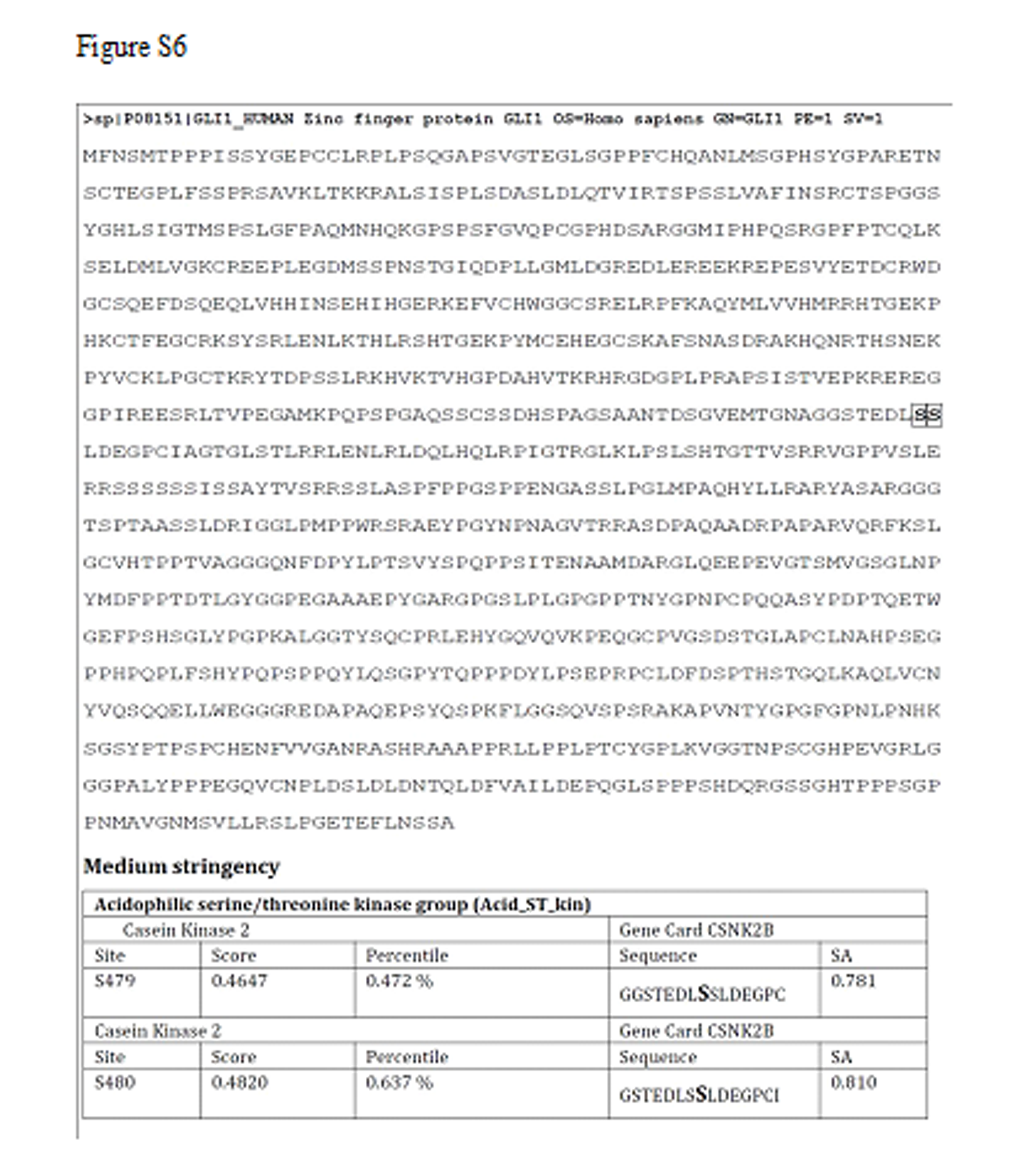

Supplement: Figure S6 — The phosphorylation prediction results of human Gli1 with CK2. Two CK2 phosphorylation sites (indicated in box) in Gli1 (NCBI Reference Sequence: AAM13391.1) were predicted using Scansite 2.0 with medium stringency. (TIF) [file pone.0038996.s006.tif]
