# Supplementary material for: Inhibition of CK2α Down-Regulates Hedgehog/Gli Signaling Leading to a Reduction of a Stem-Like Side Population in Human Lung Cancer Cells
Source: PLoS One. 2012 Jun 29;7(6):e38996. doi: 10.1371/journal.pone.0038996 (PMC3387212; doi:10.1371/journal.pone.0038996)
Supplement: Text S1 — Supplementary materials and methods. (DOC) [file pone.0038996.s007.doc]

Text S1. Supplementary Materials and Methods 
SiRNA and Plasmid DNA Transfection. CK2á, CK2á' and CK2â-specific siRNAs (ON-TARGET plus SMARTpool) and control RNA were purchased from Thermo Scientific (Waltham, MA). In brief, cells were seeded in a 6-well plate as 105 cells/well one day before transfection, with a target of 30-50% confluency at the time of transfection. Cells were transfected with 50 nmol/L of siRNA using Lipofectamine RNAiMAX (Invitrogen, Carlsbad, CA) according to the manufacturer's protocol. Adequate inhibition of the siRNA-mediated knockdown was confirmed by RT-PCR. The pcDNA3.1-CK2á or control pcDNA3.1-LacZ plasmid vectors were then transfected into the A549 cells (0.5 ìg/ml in 24-well plate) using Lipofectamine 2000 transfection reagent (Invitrogen), according to the manufacturer's protocol. Cells were harvested for RT-PCR and Western blot or used in reporter assays at 48 hours post-transfection. 
RNA Isolation, cDNA Synthesis and Semi-quantitative RT-PCR. Isolation of RNA was performed using RNeasy Mini kit (Qiagen, Valencia, CA). Human Lung Total RNA was purchased from Applied Biosystems (Foster City, CA). Five-hundred ng of total RNA was converted into 20 ìl cDNA using iScript cDNA Synthesis Kits (Bio-Rad, Hercules, CA,) according to the manufacturer's recommendations. PCR bands were visualized under UV light and photographed.  
Real-Time RT-PCR. A total of 2 ìl of the reverse transcription reaction were used as template for real-time detection of Gli1 expression using TaqMan Technology on an Applied Biosystems 7000 sequence detection system (Applied Biosystems, Foster City, CA). Gene expression was quantitated for the tested genes (Gli1, Ptc1, Ptc2 and Cyclin E1) and endogenous control gene b-glucuronidase (GUSB) using the primer and probe sequences commercially (Applied Biosystems).   
Western Blot Analysis and Immunofluorescence Staining. Whole protein was extracted by M-PER Mammalian Protein Extraction Reagent (Thermo) from cell lines added with Phosphatase Inhibitor Cocktail Set II (Calbiochem, San Diego, CA) and Complete Protease Inhibitor Cocktails (Roche, Lewes, UK) according to manufactures' protocols. The proteins were separated on 4–15% gradient SDS–polyacrylamide gels and transferred to Immobilon-P membranes (Millipore, Bellerica, MA). The following primary antibodies were used: anti-CK2á (Millipore), anti-Gli1 (Cell Signaling, Beverly, MA), anti-ABCG2 (Millipore), and anti-GAPDH (Trevigen, Gaithersburg, MD). After being incubated with appropriate secondary antibodies, the antigen-antibody complexes were detected by using an ECL blotting analysis system (Amersham Pharmacia Biotech, Piscataway, NJ). Immunofluorescence staining was carried out. Examination was done using laser scanning confocal microscopy (LSM510, Carl Zeiss, Oakland, CA). Digital images of single confocal slices were prepared using Adobe Photoshop 6.0. 
Protein Degradation Assay. The CK2á- and control siRNA-transtected A549 cells were exposed to 50 µg/ml cycloheximide and harvested at the time points of 0 and 6 hours. Total cellular proteins were extracted and were analyzed by western blot analysis. 
